# Clinicopathological features of infiltrating lobular carcinomas comparing with infiltrating ductal carcinomas: a case control study

**DOI:** 10.1186/1477-7819-8-34

**Published:** 2010-04-27

**Authors:** Ju-Hyun Lee, Seho Park, Hyung Seok Park, Byeong-Woo Park

**Affiliations:** 1 Department of Surgery, Yonsei University College of Medicine, Seoul, Korea

## Abstract

**Background:**

Infiltrating lobular carcinoma (ILC) is the second most common type of invasive breast cancers and it has been reported to have some unique biologic and epidemiologic characteristics.

**Methods:**

Clinicopathological features of 95 patients with ILC, their relapse free survival (RFS) and overall survival (OS) were retrospectively investigated and compared with those of 3,621 patients with infiltrating ductal carcinoma-not otherwise specified (IDC-NOS) between January 1984 and December 2005.

**Results:**

ILC constitutes 2.3% of all invasive breast cancers. There were no difference between the ILC and the IDC-NOS groups regarding age at diagnosis, tumor size, nodal status, and treatment modalities except hormone therapy. The ILC group showed more estrogen receptor expression, less HER-2 expression and higher bilaterality. RFS and OS of the ILC patients were similar to those of the IDC. IDC-NOS metastasized more frequently to the lung and bone, whereas, ILC to the bone and ovary.

**Conclusions:**

The incidence of ILC was relatively low in Korean breast cancer patients. Comparing to IDC-NOS ILC showed some different features such as higher estrogen receptor expression, less HER-2 expression, higher bilaterality and preferred metastatic sites of bone and ovary. Contralateral cancers and bone and ovary evaluation should be considered when monitoring ILC patients.

## Background

Infiltrating lobular carcinoma (ILC) constitutes approximately 10% of invasive breast cancers, and it is the second common histologic type of breast cancer [1]. In Korea, the incidence of ILC is quite low, and it has been reported as 2-4% of all breast cancers [2]. Recently, the incidence of ILC in the West is relatively increasing, while that of infiltrating ductal carcinoma (IDC) remains constant [1].

It has been reported that ILC has characteristics that are different from IDC [3] such as older age onset, larger tumor size, increased propensity for multifocality and multicentricity, and higher risk of bilateral breast cancer. In terms of histologic features, ILC shows better differentiation, more frequent expression of estrogen receptor (ER)-positive and progesterone receptor (PR), and less vessel invasion. Other characteristics of ILC include lower S-phase fraction and diploid, less HER-2, P53, and epidermal growth factor receptor (EGF-R) expression [3].

While some reported that ILC patients showed better prognosis, others reported poorer prognosis compared with IDC [4]. Limitation of those studies regarding outcomes of ILC is the relatively small sample sizes. The aim of this study was to investigate the clinicopathological features and outcomes of ILC, and compare them with those of infiltrating ductal carcinoma-not otherwise specified (IDC-NOS) in Korean patients.

## Methods

We retrospectively reviewed the Severance Hospital Breast Cancer Registry Database of 4,465 breast cancer patients who had been treated at the Department of Surgery, Yonsei University of College of Medicine, Seoul, Korea, between January 1984 and December 2005. Among 4,465 cases, 4,053 were invasive breast cancers: 3,621 IDC-NOS (89.3%), 95 ILC (2.3%) and 337 other types (8.4%). Among 4,053 invasive cancers, 3,621 IDC-NOS and 95 ILC were included in this study.

The patients' medical records were collected in the Severance Hospital Breast Cancer Registry Database and reviewed to obtain data regarding general characteristics of the patients, histopathology of primary tumors, treatment modalities (surgery, chemotherapy, radiation and hormone therapy), relapse-free survival (RFS) and overall survival (OS). In cases of patients diagnosed with ILC, a specialized pathologist with extensive experience in breast pathology reviewed their pathologic slides, and in cases of patients with IDC, we reviewed these cases through previous pathologic reports. Histological diagnostic features of ILC were loosely dispersed strands of infiltrating tumor cells, often in the form of a single file, throughout the fibrous matrix, lacks of cohesion without formation of tubules or papillae, and small cells with relatively little nuclear pleomorphism [5]. We did not discriminate between classic and variant types. Tumor stages were determined based on the 6th American Joint Committee on Cancer (AJCC) criteria. Histologic types followed the World Health Organization (WHO) classification.

All immunostainings were done with formalin-fixed and paraffin-embedded tissue sections. Briefly, 5 μm-thick sections were obtained with a microtome, deparaffinized and rehydrated. After treatment with 3% hydrogen peroxide solution for 10 minutes to block endogenous peroxidases, the sections were pretreated in 10 mM citrate buffer (pH 6.0) for antigen retrieval in a microwave oven for 20 minutes. After incubation with primary antibodies against ER (clone SP1, 1:100; Thermo Scientific, Fremont, CA, USA), PR (clone PgR 636, 1:50; DAKO, Glostrup, Denmark), and HER-2 (polyclonal, 1:1500; DAKO), immunodetection was performed with biotinylated antimouse immunoglobulin, followed by peroxidase-labeled streptavidin using a labeled streptavidin biotin kit with 3,3'-diaminobenzidine chromogen as substrate. Slides were counterstained with Harris hematoxylin. Hormone receptor positivity was defined as more than 10 fmol/mg cytosol protein, or as 10% or more nuclear stain by immunohistochemistry (IHC). IHC stain results of HER-2 were scored by counting the number of cells positively stained on the membrane and expressed as a percentage of total tumor cells. HER-2 staining was scored according to the American Society of Clinical Oncology (ASCO)/College of American Pathologists (CAP) guideline [6] using the following categories: 0, no immunostaining; 1+, weak incomplete membranous staining in any proportion of tumor cells; 2+, complete membranous staining, either non-uniform or weak in at least 10% of tumor cells; and 3+, uniform intense membranous staining in >30% of tumor cells.

Associations between categorical variables were assessed using a chi-square test. The RFS time was defined as from the date of the operation to the date of first locoregional or systemic recurrence. Locoregional recurrence included relapses in the chest wall, operation scar, remnant breast, axillary lymph node, supraclavicular lymph node and internal mammary lymph node. Systemic recurrences included metastases in the lung, brain, liver, bone, ovary, kidney and other organs. The OS time was defined as the period between the date of operation and the date of death from any cause. Survival curves were plotted and estimated using the Kaplan-Meier method. Group differences in the survival time were calculated by the log-rank test. The Cox proportional hazard model was used to calculate the hazard ratio (HR) and 95% CI in the analyses of RFS and OS. A p-value less than 0.05 was considered statistically significant. SPSS for Windows (version 15.0, SPSS Inc., Chicago, IL, U.S.A.) was used for all statistical analyses.

## Results

The mean follow-up duration was 74 ± 48.19 months in all patients. The mean age at diagnosis was 47.43 ± 8.91 years in ILC patients and 47.69 ± 10.31 years in IDC-NOS patients.

The clinicopathological factors are summarized in Table 1. There was no difference in the age at diagnosis, tumor size, nodal status and TMN stage. ILC patients showed higher ER expression and less HER-2 expression than IDC-NOS patients (*P *= 0.003 and 0.038, respectively). PR expression was higher in ILC, but, there was no statistical significance. There was a higher incidence of bilateral cancer in the ILC group compared to the IDC-NOS patients (*P *= 0.013).

**Table 1:** Biological characteristics of the patients and tumors by histologic group

	Histopathology					
	**Infiltrating ductal carcinoma**		**Infiltrating lobular carcinoma**		**Total**	

**Factor**	**Number of patients** **(n = 3621)**	**%**	**Number of patients** **(n = 95)**	**%**	**Number of patients**	***P *value**

Age, years						0.069
>35	3,213	88.7	90	94.7	3,303	
≤35	406	11.3	5	5.3	413	

T stage						0.922
T1	1,585	73.8	42	44.2	1,621	
T2	1,780	49.2	49	51.6	1,825	
T3	181	5	3	3.2	184	
T4	72	2	1	1.1	73	

N stage						0.56
N0	1,912	52.9	57	60	1,969	
N1	924	25.6	22	23.2	946	
N2	459	12.7	10	10.5	469	
N3	316	8.8	6	6.3	322	

TNM stage						0.57
Stage 1	1,066	29.5	28	29.5	1,094	
Stage 2	1,703	47.2	49	51.6	1,752	
Stage 3	840	23.3	18	18.9	858	

Bilaterality						0.013
Unilateral	3,495	96.5	89	93.7	3,526	
Bilateral	126	3.5	6	6.3	132	

ER receptor						0.003
Negative	1,088	37.4	15	20.3	1,103	
Positive	1,821	62.6	59	79.7	1,880	

PR receptor						0.23
Negative	1,221	42.9	25	35.7	1,246	
Positive	1,625	57.1	45	64.3	1,670	

HER-2						0.038
Negative	1,373	70.8	46	83.6	1,419	
Positive	567	29.2	9	16.4	576	

ER = estrogen receptor; PR = progesterone receptor; HER-2 = human epidermal growth factor receptor-2.

Treatment modalities including operation methods were not different between the two groups except hormone therapy (Table 2). As ILC patients showed higher ER expression rates, adjuvant hormone therapy was more frequently given to them (*P *= 0.024)

**Table 2:** Local and systemic adjuvant therapies by histologic type

	Histopathology					
	**Infiltrating ductal carcinoma**		**Infiltrating lobular carcinoma**		**Total**	

	**Number of patients**	**%**	**Number of patients**	**%**	**Number of patients**	***P *value**

Local Tx						0.297
BCS	735	20.3	15	16	750	
Mastectomy	2,880	79.7	79	84	2,959	

Radiation Tx						0.405
None	2,188	63.8	53	59.6	2,241	
Radiation Tx	1,239	36.2	36	40.4	1,275	

Chemotherapy						0.898
None	988	28	26	27.4	1,014	
Chemotherapy	2,545	72	69	72.6	2,614	

Hormone Tx						0.024
None	1,553	45.1	31	33.3	1,584	
Hormone Tx	1,889	54.9	62	66.7	1,951	

BCS = breast conserving surgery; Tx = therapy.

**Figure 1:**
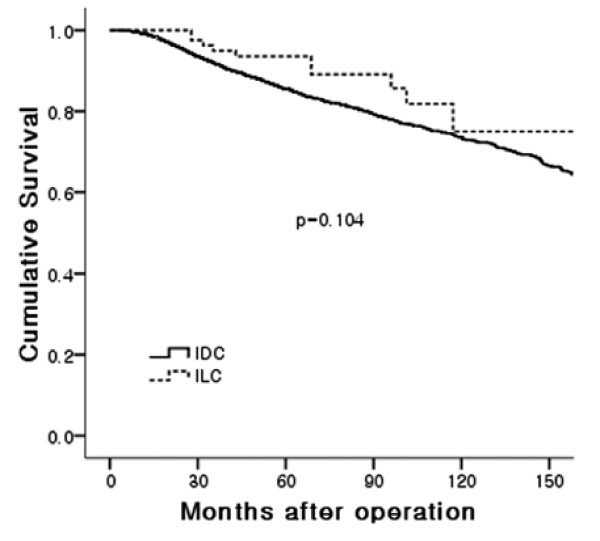
**Overall survival**.

**Figure 2:**
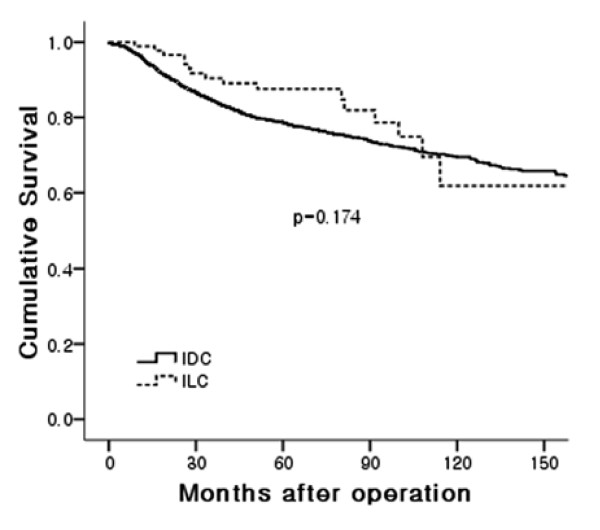
**Relapse-free survival**.

The ten-year RFS rate was 61.8% for the ILC and 69.5% for the IDC-NOS, respectively. The ten-year OS rate was 75% in ILC and 73.6% in IDC-NOS, respectively. There was no statistical significance in the RFS (relapse-free survival) and OS (overall survival) rates between the groups (Figures 1 and 2) even in stage-matched analysis (data not shown). In Table 3, recurrence patterns and frequent sites of systemic recurrences are summarized. Both groups showed similar recurrence patterns. Lung and bone were the preferred sites of metastases in the IDC-NOS group (lung: 37.5%, bone: 36.5%). In the ILC group, bone was the most frequent site of metastasis followed by ovary (bone: 72.7%, ovary: 18.2%).

**Table 3:** Recurrence patterns and distant sites of the first recurrence by histopathologic type

	Histopathology					
	**Infiltrating ductal carcinoma**		**Infiltrating lobular carcinoma**		**Total**	

	**Number of patients**	**%**	**Number of patients**	**%**	**Number of patients**	***P *value**

Pattern of recurrence						0.097
Locoregional recurrence	123	14.4	3	18.8	123	
Systemic recurrence	480	56.1	12	75.0	492	
Locoreginal recurrence and systemic recurrence	252	29.5	1	6.3	253	

Site of systemic recurrence						
Lung	264	37.5	1	9.1	254	
Brain	50	7.1	0	0	49	
Liver	105	14.9	0	0	105	
Bone	257	36.5	8	72.7	261	
Ovary	1	0.1	2	18.2	2	
Pericardium, pleura	10	1.4	0	0	8	
Contalateral SCN	4	0.6	0	0	7	
Other^a^	13	1.8	0	0	19	

Other^a ^contains opposite breast (2), contralateral axilla (1), mediastinum (1), carcinomatosis (4), adrenal gland (2), multiple lymph node (1), neck node (2). SCN = supraclavicular lymph node.

## Discussion

ILC is the second most common type of invasive breast cancers. According to the database of the National Cancer Institute from the period of 1992 up to 2001, 70% of all breast cancers were IDC, 8% were ILC, 7% were ductal/lobular type and the remaining 9% were various rare entities [1]. In American and European studies, the portion of ILC was reported to be about 10~14% [7]. However, the portion of ILC in Korea was lower than that of outside the country, and it was reported to be 2~4% [2,8]. In this analysis, ILC accounted for 2.3%, a level similar to those found in other Korean studies. Taken together, the incidence of ILC in Korea is quite lower than that of Western countries.

Comparing to patients with IDCs, patients with ILC are older than those with IDC [7,9]. However, there was no difference in the age at diagnosis between the two groups in our analysis. Low incidence of ILC and no difference in the age at diagnosis could be partly explained by different genetic or environmental factors of Korean breast cancer patients compared to those of Western countries [10] and the epidemiologic characteristics of Korean breast cancers [2]. The incidence of breast cancer in European and American women increases as they get older. In Korean women, however, the incidence of age distribution reaches the peak in their 40's, and then it declines afterwards. The most prevalent age for breast cancer in Korea is 10 years younger than that in Western countries [8].

Some studies reported that tumor size was slightly larger in the ILC group [9]. The lack of desmoplastic reaction may make the lesion impalpable and invisible, both clinically and mammographically, which could partly explain why lobular carcinomas were larger at diagnosis than IDCs [11]. However, no difference in tumor size was observed between the two groups in this study. Because of the high incidence of young breast cancer patients, the use of ultrasonography is very popular, which might in some part contribute to early detection of ILC as well as IDC in Korea.

Axillary lymph node involvement was not different between the two groups in our study. MacGrogan *et al*. [12] reported that the uniform appearance of bland tumor cells that lack cellular atypia and often have a low mitotic rate make it more difficult to correctly detect lobular cancer cells in metastatic lymph nodes. That is why nodal metastases are more often undetected in patients with ILC, and false-negative results are more frequently reported compared with ductal carcinomas [12]. Therefore, pathologists should be informed of the ILC case before interpreting the frozen slides of sentinel lymph nodes during surgery.

In agreement with other previous studies [13,14], higher rate of ER expression and lower incidence of HER2 expression were demonstrated in this study. ILC is more often found to be multifocal, multicenteric and bilateral than IDCs. Bilateral involvement has been reported to be 20~29% in lobular carcinoma [15,16]. We also observed that bilateral involvement was more common in ILC (6.3%) compared to that in IDC-NOS (3.5%) (*P *= 0.013).

Several studies reported that ILC was less often treated with breast conserving surgery than IDC [7,9]. This is attributed to the difficulty in defining tumor margins clinically or radiologically and related to higher probability of obtaining positive surgical margins due to tumor characteristics of multifocality or multicentricity [3]. Histologically ILC presents a loosely dispersed single cell pattern of growth throughout the stroma, lacks of desmoplastic reactions, and is often associated with lobular carcinoma in-situ and inflammatory cells around ducts may mimic the targetoid pattern of ILC [5,17]. These can present notable diagnostic problems, especially in frozen section or in core needle biopsy specimens. In our study, less ILC patients underwent breast conserving surgery than IDC-NOS patients did, but there was no statistical difference. Some researchers reported that breast conserving surgery in ILC patients is not associated with increased local relapse rates at 5 years when compared with mastectomy. Therefore, ILC can be treated with breast conserving surgery when clear margin can be achieved [7,18]. In terms of adjuvant therapy, both radiation therapy and chemotherapy were performed irrespective of histologic type. However, patients with ILC were more likely to undergo anti-estrogen therapy.

In terms of prognosis, some studies reported that the prognosis of ILC patients was similar to that of patients with IDC, and histologic type was not a factor affecting the prognosis [7,11]. Others reported that early prognosis for ILC was better than that for IDC, while late prognosis for ILC was worse [3]. In this analysis, RFS and OS were similar between the two groups.

The locoregional or systemic recurrence pattern of ILC was similar to that of IDC-NOS in our study. Still, the distant metastatic sites of ILC differed from those of IDC-NOS. It was observed that ILC is less likely to affect the lungs, pleura, and CNS than IDC-NOS does. By contrast, bone, peritoneum, ovary, meniges, and the gastrointestinal system were much more likely to be affected by ILC [3,19,20]. As in other reports, the current study demonstrated that bone and ovary were the preferred sites of metastasis but lung and liver were rarely affected by ILC. Difference in favoring metastatic sites by histologic types might be attributable to the microenvironments of the ovary or peritoneum which might provide growth and survival factors that favor ILC cells over IDC cells [21,22]. Some reported that loss of expression of the cell-cell adhesion molecule E-cadherin in ILC may decrease adhesiveness of cells and facilitate this type of infiltration [23]. Alternatively, cell size or shape with physical properties might favor certain microanatomical areas and that is more conductive to stopping or trapping these types of cells [19].

The limitation of our study was the HER-2 results. We defined HER-2 overexpression as 2+ or 3+ cases by HER-2 immunohistochemical testing in our study. During the periods under study approximately half of the study cohorts did not perform HER-2 immunohistochemical testing and furthermore, FISH was not a routine test for patients with 2+ results in our institution. This definition would overestimate the cases with HER-2 overexpression. However, this study is not confirmative but exploratory by retrospective review of data. Therefore, we concentrated on the sensitivity of HER-2 immunohistochemical testing. HER-2 immunohistochemical testing compared with FISH showed high sensitivity, negative predictive value, and overall accuracy when HER-2 overexpression was defined as 2+ or 3+ immunohistochemical results [24,25]. Overall HER-2 overexpression rates of our study cohorts were 28.9% (576/1995), which did not significantly different from previous report [26].

In summary, low incidence of ILC in the Korean breast cancer population might be associated with younger age onset epidemiologic characteristics of the Korean population. ILC has some different characteristics from IDC such as more frequent estrogen receptor expression, less frequent HER-2 expression, high incidence of bilaterality and bone and ovary preference of metastases. Based on the histologic and biologic characteristics, researchers should be focused on the occurrence of contralateral breast cancers and preferred sites of metastasis during follow up. In Korea, the incidence of ILC is relatively lower than that in Western. However, increase in life expectancy or patients with breast carcinoma and change to westernized life style in Korea might lead to increase the incidence of ILC. Therefore, it should be necessary to understand clinicopathological features or tumor biology of ILC and to provide appropriate diagnostic or therapeutic guidelines for ILC.

## Competing interests

The authors declare that they have no competing interests.

## Authors' contributions

JHL participated in the design of the study and performed the statistical analysis. BWP conceived of the study, and participated in its design and coordination and helped to draft the manuscript. All authors read and approved the final manuscript.
